# The effects of hyperlipidemia on rotator cuff diseases: a systematic review

**DOI:** 10.1186/s13018-018-0912-0

**Published:** 2018-08-17

**Authors:** Yang Yang, Jin Qu

**Affiliations:** 1 Department of Cardiovascular Disease, The Second Xiangya Hospital, Central South University, Changsha, 410011 People’s Republic of China; 2 Department of Sports Medicine, Key Laboratory of Organ Injury, Aging and Regenerative Medicine of Hunan Province, Xiangya Hospital, Central South University, Changsha, 410008 People’s Republic of China

**Keywords:** Hyperlipidemia, Statins, Rotator cuff diseases

## Abstract

**Background:**

Rotator cuff disease is a common condition that causes shoulder pain and functional disability. Recent studies suggested that hyperlipidemia might be associated with the development of rotator cuff disease. The objective of this study was to explore the relationship of hyperlipidemia and rotator cuff diseases.

**Methods:**

A computerized search using relevant search terms was performed in the PubMed, EMBASE, and Cochrane Library databases, as well as a manual search of reference and citation lists of the included studies. Searches were limited to studies that explored the association of hyperlipidemia and rotator cuff diseases.

**Results:**

Sixteen studies were included in this systematic review. Ten of sixteen included studies suggested an association between dyslipidemia and rotator cuff diseases, while the other six studies did not find an association. Two studies demonstrated there were an association between statins and reduced risk of developing rotator cuff diseases or decreased incidence of revision after rotator cuff repair.

**Conclusion:**

The current study suggested that there was an association between hyperlipidemia and rotator cuff diseases. Furthermore, current evidence suggested that use of statins could decrease the risk of developing rotator cuff diseases and the incidence of revision after rotator cuff repair. Future high-quality studies are highly needed to confirm these findings.

## Introduction

Rotator cuff disease is a common condition that causes shoulder pain and functional disability, with a prevalence rate of 2.8–15% [1]. Surgical repair has been a commonly accepted treatment for full-thickness rotator cuff tears. However, failure rates after arthroscopic repair have been reported up to 94% [2]. Factors affecting rotator cuff healing include patient age, tear characteristics, duration of symptoms, osteoporosis, diabetes, and smoking [3].

Hyperlipidemia is a systemic metabolic disease characterized by abnormally high levels of lipids in the blood. Hyperlipidemia has well-known impact on vascular systems and internal organs [4]. Recently, the influence of hyperlipidemia on musculoskeletal system has attracted much attention [5, 6]. In hyperlipidemia environments, lipids could accumulate within the extracellular matrix of the tendon and thus affect the mechanical properties of the tendon [7]. Several studies have explored the relationship between hyperlipidemia and rotator cuff disease [8–11]. Animal studies indicated that high levels of lipids would lead to poorer mechanical properties and adversely affect tendon-to-bone healing after surgical repair of rotator cuff tears [9–11], while clinical studies showed inconsistent results on the association between hyperlipidemia and rotator cuff disease [8, 12].

Statins, also known as hydroxy-methyl-glutaryl-coenzyme-A reductase inhibitors, are the most widely prescribed medications to treat hyperlipidemia and reduce the risk of cardiovascular diseases and related mortality [13]. Statins are known to have a potentially deleterious effect on muscle [14]. Recent studies suggested that use of statins might be associated with tendinopathy and tendon ruptures [15, 16]. In contrast, several studies demonstrated that use of statins could significantly decrease the risk of the development of rotator cuff disease in patients with hyperlipidemia [8]. In addition, the use of statins appeared to decrease the revision rate after arthroscopic rotator cuff repair [17].

The main purpose of this systematic review was to explore the relationship between hyperlipidemia and rotator cuff diseases. By the way, the potential effects of statins on rotator cuff diseases were explored in these hyperlipidemia patients.

## Materials and methods

### Search strategy

An electronic search was performed using the following databases: PubMed (until 2018/03/25), EMBASE (until 2018/03/25), and Cochrane Central Register of Controlled Trials (CENTRAL) databases (until 2018/03/25). The search algorithm was “(rotator cuff or shoulder) AND (hyperlipidemia or dyslipidemia or hypercholesterolemia or statin).” The full text was reviewed if the abstract suggested the article might be eligible. In addition to electronic database searches, the reference and citation list of each included study was reviewed to identify any potential eligible studies.

### Study selection

Eligibility criteria were (1) human studies and (2) exploring the association of hyperlipidemia and rotator cuff diseases. Exclusion criteria were (1) case reports, (2) animal studies, and (3) review articles or editorial articles.

### Data extraction

Two reviewers independently extracted data from all eligible studies with a pre-developed data extraction form. The following information was collected: where the study was conducted, when the report was published, study design, level of evidence, participants, groupings, age, sex, definition of dyslipidemia (including triglycerides (TG), total cholesterol (TC), low-density lipoprotein cholesterol (LDL-C), and high-density lipoprotein cholesterol (HDL-C)), statin use or not, primary findings, and association between hyperlipidemia and rotator cuff diseases. Any discrepancies between reviewers were resolved by consensus.

### Study quality assessment

Study quality was assessed independently by two reviewers with the MINORS (Methodological Index for Non-Randomized Studies) criteria [18]. MINORS is a validated scale, and the global ideal score is 16 for non-comparative studies and 24 for comparative studies. Any discrepancies between reviewers were resolved by consensus.

### Data analysis

A quantitative analysis was not performed because the included studies were heterogeneous with regard to study design, participants, grouping, sample size, and statistical methods. However, all eligible studies explored the association of hyperlipidemia and rotator cuff diseases. Therefore, the clinical results were only summarized in the current systematical review.

## Results

### Literature search

**Fig. 1:**
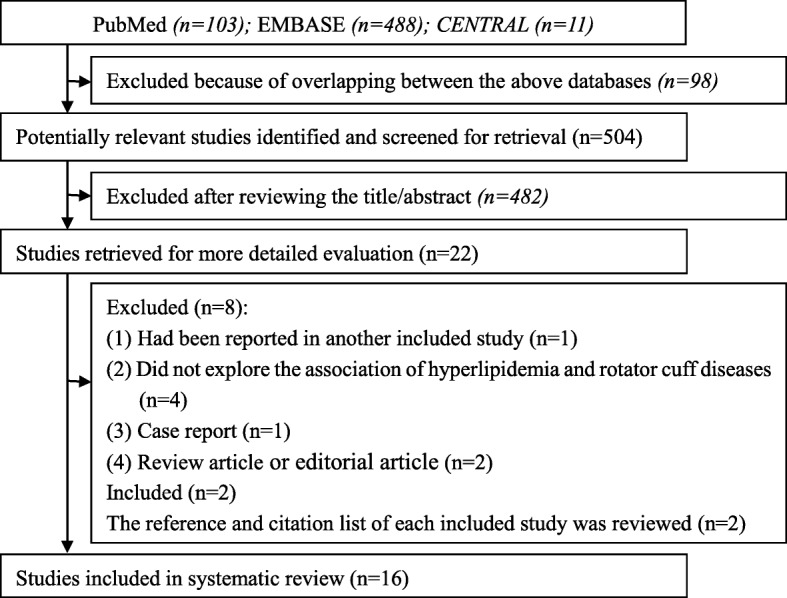
The flow diagram illustrating the search process

The literature search totally generated 602 relevant citations from the three databases (Fig. 1). After excluding the duplicates, there were 504 articles left. Subsequent review of the title/abstracts generated 22 articles that were retrieved for further evaluation. One study was excluded because the results had been reported in another included study. One study was excluded due to case reports. Four studies were excluded because the study did not explore the association of hyperlipidemia and rotator cuff diseases. Two studies were excluded because of review article or editorial article. Two studies were added after reviewing the reference and citation list of each included study [19, 20]. Finally, 16 studies were included in this systematic review [8, 12, 17, 19–31].

### Study quality and characteristics

Six studies were conducted in European countries [12, 19, 20, 22, 23, 28], six in North American countries [17, 21, 25, 26, 29, 30], and four in Asian countries [8, 24, 27, 31]. Three studies were prospective cohort study [21, 23, 25], seven were retrospective cohort study [8, 17, 20, 24, 26, 27, 29], two were case-control study [12, 31], two were cross-sectional study [19, 30], one was case series [28], and one was retrospective observational study [22]. Of the 16 studies analyzed, ten were comparative and analyzed using the 24-point scale; the remaining six non-comparative studies were analyzed on the 16-point scale. The mean MINORS score for the ten comparative studies was 19.4 ± 1.11 (maximum 24). The mean MINORS score for the six non-comparative studies was 11.5 ± 0.5 (maximum 16). The participants included varied among all eligible studies. Twelve studies only included subjects without rotator cuff surgical repair [8, 12, 19–25, 28, 30, 31], three studies only included patients with rotator cuff surgical repair [17, 26, 27], and one study included patients with surgical treatment or conservative treatment [29]. The total sample size ranged from 85 to 498,678. The percentage of male ranged from 0 to 62% in 15 studies, except one study did not report the percentage of male [25]. The age of participants was reported in 15 studies, and the average age ranged from 42.5 to 77.5 years. The main study quality and characteristics are summarized in Table 1.

**Table 1:** Study characteristics

Author	Year	Country	Design	Level of evidence	Participants	Grouping	Sample size	Sex (percent of male)	Average age (years)	MINORS
Abboud and Kim [21]	2010	USA	Prospective cohort study	Level II	Patients from an outpatient tertiary care clinic for shoulder pain necessitating surgery	Study group: with rotator cuff tears Control group: with shoulder pain but without tears	Study group, 74 Control group, 73	Study group, 59.5% Control group, 53.4%	Study group, 66.3 Control group, 67.4	21/24
Longo et al. [12]	2010	England	Case-control study	Level III	Patients operated at one institution	Study group: arthroscopic repair of a rotator cuff tear Control group: arthroscopic Meniscectomy for a meniscal tear	Study group, 120 Control group,120	Study group, 37.5% Control group, 37.5%	Control group, 64.9Study group, 63.9	18/24
Abate et al. [19]	2014	Italy	Cross-sectional study	Level IV	Female patient with lower limb diseases	Group 1: older than 44 years and with regular menstrual cycles Group 2: postmenopause 2–7 years	Group 1, 110 Group 2, 122	Total, 0%	Group 1, 46.8 Group 2, 52.7	19/24
Oliva et al. [22]	2014	Italy	Retrospective observational study	Level IV	Patients with non-traumatic rotator cuff tear	Not available	Total, 441	Total, 37%	Total, 61.1	12/16
Djerbi et al. [23]	2015	France	Prospective cohort study	Level II	Patients operated in the same orthopedic unit	Study group: patients undergoing arthroscopic rotator cuff repair Control group: operated on other parts but not shoulder	Study group, 206 Control group, 100	Study, 60% Control, 55%	Study, 57.8 Control, 59.4	21/24
Lin et al. [8]	2015	China	Retrospective cohort study	Level III	Randomly selected from national health research database	Not available	Total, 498,678	Total, 50.8%	Total, 48.8	12/16
Davis et al. [25]	2016	USA	Prospective cohort study	Level III	Patients undergoing shoulder surgery	Study group: rotator cuff tear requiring a repair Control group: with intact rotator cuff	Study group, 40 Control group, 37	Not available	Study, 57.5 Control, 53.7	21/24
Kim et al. [24]	2016	Korea	Retrospective cohort study,	Level III	Supraspinatus tendinopathy with or without tear	Study group: with dyslipidemia Control group: without dyslipidemia	Study group, 49 Control group, 50	Study group, 36.7% Control group, 38%	Study group, 58.1 Control group, 55.6	19/24
Abate et al. [28]	2017	Italy	Case series	Level IV	Patients with non-traumatic and symptomatic rotator cuff tear	Group 1: monolateral rotator cuff tear Group 2: bilateral rotator cuff tear	Group 1, 111 Group 2, 69	Group 1, 35.2% Group 2, 37.7%	Group 1, 59.2 Group 2, 63.6	19/24
Applegate et al. [30]	2017	USA	Cross-sectional study	Level IV	Workers were recruited from 17 diverse production facilities	Not available	Total, 1226	Total, 34.3%	Total, 42.1	11/16
Lai et al. [29]	2017	USA	Retrospective design	Level III	Patients with rotator cuff tears for surgery repair or physical therapy	Not available	Total, 135	Total, 57.8%	Total, 61.1	12/16
Juge et al. [20]	2017	France	Retrospective cohort study	Level III	Patients with shoulder osteoarthritis	Group 1: rotator cuff -related osteoarthritis Group 2: primary shoulder osteoarthritis	Group 1, 48 Group 2, 99	Group 1, 35.4% Group 2, 29.3%	Group 1, 72.4 Group 2, 77.5	18/24
Kim et al. [27]	2017	Korea	Retrospective cohort design	Level III	Consecutive patients who underwent arthroscopic rotator cuff repair	Not available	Total, 180	Total, 46.7%	Total, 60.4	11/16
Garcia et al. [26]	2017	USA	Retrospective cohort design	Level III	Patients with arthroscopic rotator cuff repair.	Study group: with hyperlipidemia Control group: without hyperlipidemia	Study group, 33 Control group, 52	Total, 62.8%	Total, 62.1	19/24
Cancienne et al. [17]	2017	USA	Retrospective cohort design	Level III	Patients with primary arthroscopic rotator cuff from postoperative database	Not available	Total, 30638	Total, 51.5%	Between 40 and 85 years	11/16
Yamamoto et al. [31]	2017	Japan	Case-control study	Level III	Consecutive patients with symptomatic rotator cuff tears	Group 1: tear progression Group 2: tear nonprogression	Group 1, 82 Group 2, 92	Group 1, 60% Group 2, 55.4%	Group 1, 68 Group 2, 65.9	19/24

### Dyslipidemia

The definition of dyslipidemia varied among studies. Three studies did not specify the definition of dyslipidemia and the kinds of lipids [8, 22, 31]. Nine studies determined the dyslipidemia only by the levels of lipids [12, 17, 19, 21, 24, 25, 27, 29, 30]. Three studies diagnosed the dyslipidemia based on medical history, cholesterol-lowering drugs, and the levels of lipids [20, 23, 28]. One study defined the dyslipidemia by the primary care physician who was currently treating each patient, not by the levels of lipids [26] (Table 2).

**Table 2:** Dyslipidemia, cholesterol-lowering medications and main findings

Author	Dyslipidemia	Cholesterol-lowering medications	Primary findings	Association
Abboud and Kim [21]	TC, TG, LDL-C, and HDL-C were measured, but the definition was not specified	Participants with cholesterol lowering medications were included but not analyzed	TC, TG, and LDL-C concentrations of the patients with rotator cuff tendon tears were significantly higher than the control group. The high-density lipoprotein cholesterol showed a trend to being lower than the control group.	Yes
Longo et al. [12]	TC > 6.2 mmol/L, LDL-C > 5.2 mmol/L, TG > 4.5 mmol/L	Participants with statins use were excluded	There was no statistically significant difference in serum TG and TC concentration.	No
Abate et al. [19]	TC, TG, and HDL-C were measured, but the definition was not specified	Not specified	High TG and low HDL-C were associated with an increased risk of asymptomatic rotator cuff tears. This was not statistically significant with TC.	Yes
Oliva et al. [22]	Hypercholesterolemia, but the definition and kinds of lipids were not specified	Records of cholesterol-lowering medications were retrieved but not analyzed	High proportions of patients with non-traumatic rotator cuff tears had hypercholesterolemia. High portions of patients with hypercholesterolemia took cholesterol-lowering medications.	Yes
Djerbi et al. [23]	TG > 1.50 g/L, LDL-C > 1.60 g/L, HDL-C > 0.40 g/L or if the patient was currently taking cholesterol-lowering drugs	Participants with cholesterol lowering medications were included but not analyzed	Patients with dyslipidemia had significantly higher odds ratio of rotator cuff tears.	Yes
Lin et al. [8]	Hyperlipidemia, but the definition and kinds of lipids were not specified	Records of statin prescriptions were retrieved and analyzed	Hyperlipidemia was an independent risk factor for rotator cuff disease development. An increased risk also existed in patients with hyperlipidemia with/without statin use. Statin use was associated with a lower risk of developing rotator cuff diseases when compared with no statin use.	Yes
Davis et al. [25]	TC, TG, HDL-C, and non-HDL-C were measured, but the definition was not specified	Patients with prescription medication for hypercholesterolemia were excluded	There were no significant differences in any lipid values between patients with rotator cuff and those without a tear	No
Kim et al. [24]	TC ≥ 240 mg/dL; HDL-C < 40 mg/dL in men or 50 mg/dL in women and LDL-C > 160 mg/dL; TG > 200 mg/dL.	Participants with lipid-lowering medications were excluded	Rotator cuff tears were more frequent in the hyperlipidemia group although statistical analysis showed no significant difference. Patients with hyperlipidemia had significantly less improvement in pain level.	Yes
Abate et al. [28]	Diagnosed on the basis of history, drugs assumption and recent (< 3 months) blood evaluations. The kinds of lipids were not defined	Records of statin prescriptions were recorded and analyzed	There was no association of bilateral rotator cuff tears with hypercholesterolemia and statin therapy	No
Applegate et al. [30]	TC ≥ 200 mg/dL	Not specified	Hypercholesterolemia was statistically associated with glenohumeral joint pain, but not rotator cuff tendinopathy.	No
Lai et al. [29]	TC ≥ 200 mg/dL, TG ≥ 150 mg/dL, LDL-C ≥ 130 mg/dL, HDL-C < 40 mg/dL	Not specified	Dyslipidemia might decrease the improvement of patient-reported outcomes in patients undergoing treatment for rotator cuff tears. High triglycerides and low HDL might have the most impact.	Yes
Juge et al. [20]	Known status and/or use of lipid-lowering agents, and/or abnormal available dosage of triglycerides or cholesterol levels	Patients with use of lipid-lowering agents was included but not analyzed	There were no significant difference in the rate of dyslipidemia between rotator cuff-related osteoarthritis and primary shoulder osteoarthritis	No
Kim et al. [27]	TC > 240 mg/dL	Not specified	BMI, dyslipidemia, and fatty infiltration of the infraspinatus were considered significant risk factors for retear.	Yes
Garcia et al. [26]	Cholesterol levels were not used. Defined by the primary care physician who was currently treating each patient	All patients with hyperlipidemia took a statin medication. Prescriptions for statins were identified and analyzed	HL patients had a significantly higher risk of retear after arthroscopic rotator cuff repair. Type and dosage of statin medication did not significantly affect the incidence of retear.	Yes
Cancienne et al. [17]	TC≥ 240 mg/dL, TG ≥ 200 mg/dL, LDL-C ≥ 160 mg/dL	Prescriptions for statins were identified and analyzed	There was significant association between moderate and high perioperative total cholesterol and LDL levels and the rate of revision rotator cuff surgery. Use of statin lipid-lowering agents decreased the need for revision rotator cuff surgery.	Yes
Yamamoto et al. [31]	Hypercholesterolemia, but the definition and kinds of lipids were not specified	Not specified	Hypercholesterolemia was not significantly correlated with tear progression	No

*TC* total cholesterol, *LDL-C* low-density lipoprotein cholesterol, *HDL-C* high-density lipoprotein cholesterol, *TG* triglyceride

### Cholesterol-lowering medications

Five studies did not specify the use of cholesterol-lowering medications [19, 27, 29–31]. Three studies excluded these patients with cholesterol-lowering medications [12, 24, 25]. Four studies included these patients with cholesterol-lowering medications, but did not analyze the data [20–23]. Four studies included these patients with statins and analyzed the association of statins and rotator cuff diseases [8, 17, 26, 28] (Table 2).

### Hyperlipidemia and rotator cuff diseases

Significant heterogeneity among these studies precluded the possibility of pooling the data (Table 2). Ten of 16 included studies suggested an association between dyslipidemia and rotator cuff diseases [8, 17, 19, 21–24, 26, 27, 29]. Six of the ten studies only included participants with or without rotator cuff tears [8, 19, 21–24], three only included participants with rotator cuff repair [17, 26, 27], and one included patients with rotator cuff tears for surgery repair or physical therapy [29]. Abboud and Kim [21] showed that TC, TG, and LDL-C concentrations of the patients with rotator cuff tears were significantly higher than the control group. The HDL-C concentration showed a trend to being lower than the control group. Abate et al. [19] indicated that high TG and low HDL-C were associated with an increased risk of asymptomatic rotator cuff tears. Oliva et al. [22] found a large proportion of patients with non-traumatic rotator cuff tears had hypercholesterolemia. Djerbi et al. [23] indicated that dyslipidemia had a significant effect on the prevalence of rotator cuff tears. Lin et al. [8] conducted a large retrospective cohort study with 498,678 participants and demonstrated that hyperlipidemia was an independent risk factor for rotator cuff disease development and an increased risk existed in patients with hyperlipidemia with/without statin use. Kim et al. [24] indicated that rotator cuff tears were more frequent in the hyperlipidemia group although statistical analysis showed no significant difference. Patients with hyperlipidemia had significantly less improvement in pain level.

As for patients with rotator cuff repair, Kim et al. [27] conducted a retrospective cohort study and demonstrated that BMI, dyslipidemia, and fatty infiltration of the infraspinatus were significant risk factors for retear after rotator cuff repair. The study by Garcia et al. [26] indicated that patients with hyperlipidemia had a significantly higher risk of retear after arthroscopic rotator cuff repair. Cancienne et al. [17] performed a retrospective cohort study with 30,638 patients and demonstrated that there was a significant association between moderate and high perioperative TC and LDL-C levels and the rate of revision rotator cuff surgery. The study by Lai et al. [29] included patients with rotator cuff tears for surgery repair or physical therapy and suggested that dyslipidemia might decrease the improvement of patient-reported outcomes in patients undergoing treatment for rotator cuff tears. High triglycerides and low HDL might have the most impact.

In contrast, six of 16 studies did not find an association between dyslipidemia and rotator cuff diseases [12, 20, 25, 28, 30, 31]. The study by Longo et al. [12] indicated no statistically significant difference in serum TG and TC concentration. Abate et al. [19] compared the prevalence of hypercholesterolemia in subjects with bilateral and monolateral rotator cuff tears and did not find an association of bilateral rotator cuff tears with hypercholesterolemia. The study by Applegate et al. [30] showed that hypercholesterolemia was statistically associated with glenohumeral joint pain, but not with rotator cuff tendinopathy. Davis et al. [25] evaluated the serum and synovial lipid profiles in patients with and without rotator cuff tear and found that there were no significant differences in any lipid values between patients with rotator cuff and those without a tear. In addition, Juge et al. [20] performed a retrospective study and indicated that there was no significant difference in the rate of dyslipidemia between rotator cuff-related osteoarthritis and primary shoulder osteoarthritis. Yamamoto et al. [31] explored the risk factors of symptomatic rotator cuff tear progression and showed that hypercholesterolemia was not significantly correlated with tear progression.

### Statins and rotator cuff diseases

Five studies did not specify whether participants with statins were included or excluded [19, 27, 29–31]. Three studies excluded the participants with statins [12, 24, 25]. Four studies included participants with statins but did not analyze these data [20–23]. Only four of 14 studies explored the association between statins and rotator cuff diseases [8, 17, 26, 28]. Two studies demonstrated there were an association between statins and the risk of developing rotator cuff diseases and the incidence of revision after rotator cuff repair [8, 17]. The study by Lin et al. [8] demonstrated that statin use was associated with a lower risk of developing rotator cuff diseases when compared with no statin use. The study by Cancienne et al. [17] indicated that use of statin lipid-lowering agents decreased the need for revision rotator cuff surgery. However, in the study by Garcia et al. [26], all patients with hyperlipidemia took a statin medication. Type and dosage of statin medication did not significantly affect the incidence of retear. Abate et al. [28] explored the effect of statins on bilateral and monolateral rotator cuff tears and did not find an association of bilateral rotator cuff tears with statins.

## Discussion

The main finding of the systematic review was that there was an association between hyperlipidemia and rotator cuff diseases. Furthermore, current evidence suggested that use of statins could decrease the risk of developing rotator cuff diseases and the incidence of revision after rotator cuff repair.

Hyperlipidemia is a systemic metabolic disease characterized by abnormally high levels of lipids in the blood. Hyperlipidemia has well-known impact on vascular systems and internal organs. The effects of hyperlipidemia on tendon disorders have been an area of emerging research. In hyperlipidemia environments, lipids could accumulate within the extracellular matrix of the tendon and thus affect the mechanical properties of the tendon. Several studies have explored the relationship between hyperlipidemia and tendinopathy. Animal studies indicated that high levels of lipids would lead to poorer mechanical properties. Beason et al. [32] explored the effects of hyperlipidemia on tendon biomechanics in a mouse model and demonstrated that hyperlipidemia could increase the likelihood of tendon tears. In another studies by Beason et al. [33], the authors harvested supraspinatus tendons from normal and high-cholesterol mice, rats, and monkeys and demonstrated that hypercholesterolemia increased supraspinatus tendon stiffness and elastic modulus across multiple species. The findings of the current systematic review strengthened the association of hyperlipidemia and tendinopathy, specifically as it related to rotator cuff diseases.

Animal studies suggested that hyperlipidemia might adversely affect tendon-to-bone healing after surgical repair of rotator cuff tears. Chung et al. [9] explored the effect of hyperlipidemia on fatty infiltration and tendon-to-bone healing in a rabbit model and demonstrated that hyperlipidemia had a deleterious effect on fatty infiltration and tendon-to-bone healing, and lowering hyperlipidemia with administration of simvastatin could reverse these harmful effects. The authors suggested that hyperlipidemia should be controlled during the perioperative period of rotator cuff repair. Beason et al. [34] evaluated the effect of hyperlipidemia on tendon-to-bone healing in a rat rotator cuff repair model and demonstrated that tendon-to-bone healing were adversely affected by hyperlipidemia. In the current systematic review, three studies explored the relationship between hyperlipidemia and the incidence of retear or revision after rotator cuff repair [17, 26, 27]. These studies consistently concluded that hyperlipidemia adversely affected the healing after surgical repair of rotator cuff tears. Better perioperative lipid control could reduce the incidence of retear and improve clinical outcomes after arthroscopic rotator cuff repair.

Statins are the most widely prescribed medications to treat hyperlipidemia and reduce the risk of cardiovascular diseases and related mortality. Recent clinical study suggested that use of statin could significantly decrease the risk of the development of rotator cuff disease in patients with hyperlipidemia. Tucker and Soslowsky [10] determined the effects of simvastatin on rat supraspinatus tendon mechanical and histological properties in a diet-induced hypercholesterolemia model and concluded that simvastatin treatment did not negatively affect tendon properties and therefore patients prescribed simvastatin might not experience tendon damage. However, Abate et al. [28] explored the effect of statins on bilateral and monolateral rotator cuff tears and did not find an association of bilateral rotator cuff tears with statins.

The effect of statins on the healing after rotator cuff repair was widely explored [11, 17]. Muscle atrophy, fibrosis, and fatty infiltration of the supraspinatus muscle usually occurred following rotator cuff tears. Statins were demonstrated to have anti-inflammatory and antifibrotic effects in many tissues and thus might protect muscles from atrophy, fibrosis, and fatty infiltration [35]. Davis et al. [36] indicated that simvastatin could reduce the incidence of muscle weakness and fibrosis after chronic rotator cuff tears. Dolkart et al. [37] indicated that atorvastatin could enhance tendon healing after rotator cuff repairs by stimulating tenocyte proliferation, migration, and adhesion via increased COX-2 activity and autocrine/paracrine PGE2 signaling. Furthermore, the study by Cancienne et al. [17] also indicated that use of statin lipid-lowering agents decreased the need for revision rotator cuff surgery. Although these studies have showed positive effects of statin use on the healing after rotator cuff repair, different results have been reported. Deren et al. [11] investigated whether local and systemic administration of simvastatin increased tendon-bone healing in a rat rotator cuff repair model and indicated that the use of systemic and local simvastatin offered no benefit. In addition, Garcia et al. [26] showed that type and dosage of statin medication did not significantly affect the incidence of retear after rotator cuff repair. The effect of statins on the healing after rotator cuff repair should be explored in more high-quality clinical trials.

Statins were reported to have potentially deleterious effect on muscle and tendon. Use of statin was associated with myalgia, muscle injury, increase in creatine kinase, and even rarer rhabdomyolysis [38]. Abd et al. [39] estimated that 10–15% of statin users would develop statin-related muscle side effects ranging from mild myalgia to more severe muscle symptoms, with significant creatine kinase elevations. Use of statins has also been demonstrated to be associated with certain tendinopathy and tendon ruptures, especially of the Achilles, quadriceps, and distal biceps tendons [15]. Marie et al. [40] conducted a retrospective study and found that tendinopathy more often occurred within the first year after statin initiation. The authors suggested that prescribers should be aware of tendinous complications related to statins. Beri et al. [41] performed a case-control study and indicated that there was no overall association between statin use and tendon rupture. While subgroup analysis suggested that women with tendon rupture were more likely to be on statins. Savvidou et al. [42] retrospectively reviewed 104 patients with distal biceps tendon rupture and concluded that there was a trend of association of spontaneous distal biceps tendon ruptures with statin administration. Although the relationship between statins and tendinous complications was not confirmed, the benefits of statins on rotator cuff diseases needed to be balanced with the potential adverse effects.

There are several limitations in the systematic review. Firstly, there was a significant heterogeneity among the included studies on study design, participants, grouping, sample size, and statistical methods. Significant heterogeneity among these studies precluded the possibility of pooling the data. Secondly, the definition of dyslipidemia varied among included studies. Different kinds of lipids (TC, TG, LDL-C, and HDL-C) had different effects on tissues. However, several included studies did not report the fractionated lipid levels. Furthermore, several studies determined the dyslipidemia by the levels of lipids, while several studies diagnosed the dyslipidemia based on medical history, cholesterol-lowering drugs, and the levels of lipids. Differently, Garcia et al. [26] defined the dyslipidemia by the primary care physician who was currently treating each patient, not by the levels of lipids. Thirdly, several studies included patients with statins but did not explore the effect of statins. Statin medications could artificially lower the levels of lipids. It was unknown whether artificially lowering levels of lipids had affected the results. Fourthly, there are many risk factors, including patient age, high BMI, diabetes mellitus, dyslipidemia, and smoking, which may affect the development of rotator cuff tears and the healing process of rotator cuff repair. Certain older participants may have all or most of these risk factors. It was therefore hard to separate these factors as definite independent factors of rotator cuff diseases.

## Conclusions

The current study suggested that there was an association between hyperlipidemia and rotator cuff diseases. Furthermore, current evidence suggested that use of statins could decrease the risk of developing rotator cuff diseases and the incidence of revision after rotator cuff repair. Future high-quality studies are highly needed to confirm the findings of this review.

## Abbreviations

COX-2: Cyclooxygenase-2
HDL-C: High-density lipoprotein cholesterol
LDL-C: Low-density lipoprotein cholesterol
PGE2: Prostaglandin E2
TC: Total cholesterol
TG: Triglycerides
